# 3-Cyclo­hexyl­sulfonyl-5-isopropyl-2-methyl-1-benzofuran

**DOI:** 10.1107/S1600536811007215

**Published:** 2011-03-02

**Authors:** Hong Dae Choi, Pil Ja Seo, Byeng Wha Son, Uk Lee

**Affiliations:** a Department of Chemistry, Dongeui University, San 24 Kaya-dong Busanjin-gu, Busan 614-714, Republic of Korea; b Department of Chemistry, Pukyong National University, 599-1 Daeyeon 3-dong, Nam-gu, Busan 608-737, Republic of Korea

## Abstract

In the title compound, C_18_H_24_O_3_S, the cyclo­hexyl ring adopts a chair conformation. In the crystal, mol­ecules are linked through weak non-classical inter­molecular C—H⋯O hydrogen bonds and C—H⋯π inter­actions.

## Related literature

For the pharmacological activity of benzofuran compounds, see: Aslam *et al.* (2006 ▶); Galal *et al.* (2009 ▶); Khan *et al.* (2005 ▶). For natural products with benzofuran rings, see: Akgul & Anil (2003 ▶); Soekamto *et al.* (2003 ▶). For structural studies of related 3-aryl­sulfonyl-5-isopropyl-2-methyl-1-benzofuran derivatives, see: Choi *et al.* (2008 ▶, 2010 ▶).
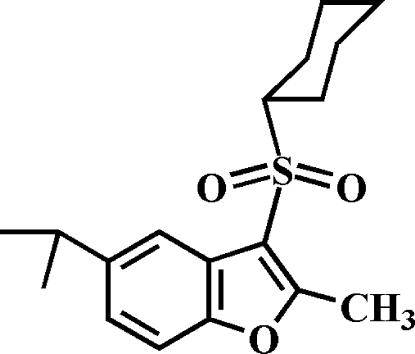


## Experimental

### 

#### Crystal data

C_18_H_24_O_3_S
*M* *_r_* = 320.44
Monoclinic, 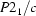

*a* = 5.7117 (1) Å
*b* = 23.5045 (4) Å
*c* = 12.7980 (2) Å
β = 102.080 (1)°
*V* = 1680.09 (5) Å^3^
*Z* = 4
Mo *K*α radiation
μ = 0.20 mm^−1^
*T* = 173 K
0.26 × 0.25 × 0.22 mm

#### Data collection

Bruker SMART APEXII CCD diffractometer
Absorption correction: multi-scan (*SADABS*; Bruker, 2009 ▶) *T*_min_ = 0.949, *T*_max_ = 0.957
15706 measured reflections
3870 independent reflections
3124 reflections with *I* > 2σ(*I*)
*R*_int_ = 0.034

#### Refinement

*R*[*F* ^2^ > 2σ(*F* ^2^)] = 0.043
*wR*(*F* ^2^) = 0.118
*S* = 1.05
3870 reflections
202 parameters
H-atom parameters constrained
Δρ_max_ = 0.23 e Å^−3^
Δρ_min_ = −0.49 e Å^−3^

### 

Data collection: *APEX2* (Bruker, 2009 ▶); cell refinement: *SAINT* (Bruker, 2009 ▶); data reduction: *SAINT*; program(s) used to solve structure: *SHELXS97* (Sheldrick, 2008 ▶); program(s) used to refine structure: *SHELXL97* (Sheldrick, 2008 ▶); molecular graphics: *ORTEP-3* (Farrugia, 1997 ▶) and *DIAMOND* (Brandenburg, 1998 ▶); software used to prepare material for publication: *SHELXL97*.

## Supplementary Material

Crystal structure: contains datablocks global, I. DOI: 10.1107/S1600536811007215/rk2267sup1.cif

Structure factors: contains datablocks I. DOI: 10.1107/S1600536811007215/rk2267Isup2.hkl

Additional supplementary materials:  crystallographic information; 3D view; checkCIF report

## Figures and Tables

**Table 1:** Hydrogen-bond geometry (Å, °) *Cg* is the centroid of the C2–C7 benzene ring.

*D*—H⋯*A*	*D*—H	H⋯*A*	*D*⋯*A*	*D*—H⋯*A*
C13—H13⋯O2^i^	1.00	2.33	3.3030 (19)	165
C14—H14*A*⋯O3^ii^	0.99	2.58	3.424 (2)	143
C10—H10*C*⋯*Cg*^i^	0.98	2.71	3.548 (2)	144

Symmetry codes: (i) 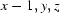; (ii) 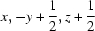.

**Fig. 1.**
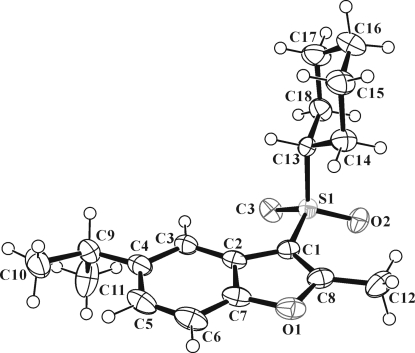
The molecular structure of the title compound with the atom numbering scheme. Displacement ellipsoids are drawn at the 50% probability level. H atoms are presented as a small spheres of arbitrary radius.

**Fig. 2.**
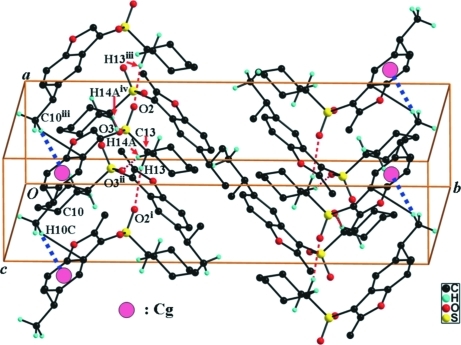
A view of the C—H···O and C—H···π interactions (dotted lines) in the crystal structure of the title compound. Symmetry codes: (i) x-1 , y, z; (ii) x, -y+1/2, z+1/2 (iii) x+1, y, z; (iv) x, -y+1/2, z-1/2.

## supplementary crystallographic information

### Comment

Many compounds having a benzofuran skeleton exhibit diverse pharmacological properties such as antifungal, antitumor and antiviral, and antimicrobial activities (Aslam *et al.*, 2006; Galal *et al.*, 2009; Khan *et al.*, 2005). These compounds occur in a wide range of natural products (Akgul & Anil, 2003; Soekamto *et al.*, 2003). As a part of our ongoing study of the substituent effect on the solid state structures of 3-arylsulfonyl-5-isopropyl-2-methyl-1-benzofuran analogues (Choi *et al.*, 2008, 2010), we report herein the molecular and crystal structures of the title compound.

In the title molecule (Fig. 1), the benzofuran unit is essentially planar, with a mean deviation of 0.009 (1) Å from the least-squares plane defined by the nine constituent atoms. The cyclohexyl ring is in the chair form. The molecular packing (Fig. 2) is stabilized by weak non-classical intermolecular C–H···O hydrogen bonds; the first one between a cyclohexyl H atom and the oxygen of the O═S═O unit (Table 1; C13—H13···O2^i^), the second one between a cyclohexyl H atom and the oxygen of the O═S═O unit (Table 1; C14—H14A···O3^ii^). The crystal packing (Fig. 2) is further stabilized by intermolecular C–H···π interactions between a methyl H atom of the isopropyl group and the benzene ring.

### Experimental

The 3-chloroperoxybenzoic acid (77%, 560 mg, 2.5 mmol) was added in small portions to a stirred solution of 3-cyclohexylsulfanyl-5-isopropyl-2-methyl-1-benzofuran (346 mg, 1.2 mmol) in dichloromethane (40 mL) at 273 K. After being stirred at room temperature for 6h, the mixture was washed with saturated sodium bicarbonate solution and the organic layer was separated, dried over magnesium sulfate, filtered and concentrated at reduced pressure. The residue was purified by column chromatography (hexane–ethyl acetate, 4:1 v/v) to afford the title compound as a colourless solid [yield 78%, m.p. 467–468 K; *R*_f_ = 0.66 (hexane–ethyl acetate, 4:1 v/v)]. Single crystals suitable for X-ray diffraction were prepared by slow evaporation of a solution of the title compound in acetone at room temperature.

### Refinement

All H atoms were positioned geometrically and refined using a riding model, with C—H = 0.95Å for aryl, 1.00Å for methine, 0.99Å for methylene and 0.98Å for methyl H atoms, respectively. *U*_iso_(H) = 1.2*U*_eq_(C) for aryl, methine and methylene, and 1.5*U*_eq_(C) for methyl H atoms.

### Crystal data

**Table:**

C_18_H_24_O_3_S	*F*(000) = 688
*M**_r_* = 320.44	*D*_x_ = 1.267 Mg m^−^^3^
Monoclinic, *P*2_1_/*c*	Melting point = 467–468 K
Hall symbol: -P 2ybc	Mo *K*α radiation, λ = 0.71073 Å
*a* = 5.7117 (1) Å	Cell parameters from 7210 reflections
*b* = 23.5045 (4) Å	θ = 2.4–27.3°
*c* = 12.7980 (2) Å	µ = 0.20 mm^−^^1^
β = 102.080 (1)°	*T* = 173 K
*V* = 1680.09 (5) Å^3^	Block, colourless
*Z* = 4	0.26 × 0.25 × 0.22 mm

### Data collection

**Table:**

Bruker SMART APEXII CCD diffractometer	3870 independent reflections
Radiation source: rotating anode	3124 reflections with *I* > 2σ(*I*)
graphite multilayer	*R*_int_ = 0.034
Detector resolution: 10.0 pixels mm^-1^	θ_max_ = 27.5°, θ_min_ = 1.7°
φ and ω scans	*h* = −7→7
Absorption correction: multi-scan (*SADABS*; Bruker, 2009)	*k* = −22→30
*T*_min_ = 0.949, *T*_max_ = 0.957	*l* = −16→16
15706 measured reflections	

### Refinement

**Table:**

Refinement on *F*^2^	Primary atom site location: structure-invariant direct methods
Least-squares matrix: full	Secondary atom site location: difference Fourier map
*R*[*F*^2^ > 2σ(*F*^2^)] = 0.043	Hydrogen site location: inferred from neighbouring sites
*wR*(*F*^2^) = 0.118	H-atom parameters constrained
*S* = 1.05	*w* = 1/[σ^2^(*F*_o_^2^) + (0.0595*P*)^2^ + 0.3753*P*] where *P* = (*F*_o_^2^ + 2*F*_c_^2^)/3
3870 reflections	(Δ/σ)_max_ = 0.001
202 parameters	Δρ_max_ = 0.23 e Å^−^^3^
0 restraints	Δρ_min_ = −0.49 e Å^−^^3^

### Special details

**Table:**

Geometry. All s.u.'s (except the s.u. in the dihedral angle between two l.s. planes) are estimated using the full covariance matrix. The cell s.u.'s are taken into account individually in the estimation of s.u.'s in distances, angles and torsion angles; correlations between s.u.'s in cell parameters are only used when they are defined by crystal symmetry. An approximate (isotropic) treatment of cell s.u.'s is used for estimating s.u.'s involving l.s. planes.
Refinement. Refinement of *F*^2^ against ALL reflections. The weighted *R*-factor w*R* and goodness of fit *S* are based on *F*^2^, conventional *R*-factors *R* are based on *F*, with *F* set to zero for negative *F*^2^. The threshold expression of *F*^2^ > 2σ(*F*^2^) is used only for calculating *R*-factors(gt) etc. and is not relevant to the choice of reflections for refinement. *R*-factors based on *F*^2^ are statistically about twice as large as those based on *F*, and *R*-factors based on ALL data will be even larger.

### Fractional atomic coordinates and isotropic or equivalent isotropic displacement parameters (Å^2^)

**Table:**

	*x*	*y*	*z*	*U*_iso_*/*U*_eq_	
S1	0.79640 (7)	0.253935 (16)	0.35223 (3)	0.02755 (13)	
O1	0.8519 (2)	0.14326 (5)	0.58586 (9)	0.0413 (3)	
O2	1.0380 (2)	0.27516 (5)	0.37375 (10)	0.0411 (3)	
O3	0.6959 (2)	0.23391 (5)	0.24608 (9)	0.0377 (3)	
C1	0.7751 (3)	0.19902 (6)	0.44030 (12)	0.0296 (3)	
C2	0.5856 (3)	0.15696 (6)	0.42805 (12)	0.0304 (3)	
C3	0.3829 (3)	0.14321 (7)	0.35150 (13)	0.0327 (4)	
H3	0.3395	0.1653	0.2883	0.039*	
C4	0.2436 (3)	0.09669 (7)	0.36817 (14)	0.0367 (4)	
C5	0.3105 (4)	0.06562 (7)	0.46338 (15)	0.0444 (5)	
H5	0.2137	0.0343	0.4750	0.053*	
C6	0.5089 (4)	0.07834 (7)	0.54037 (15)	0.0448 (5)	
H6	0.5514	0.0567	0.6042	0.054*	
C7	0.6431 (3)	0.12389 (7)	0.52048 (13)	0.0366 (4)	
C8	0.9275 (3)	0.18916 (7)	0.53575 (13)	0.0353 (4)	
C9	0.0319 (3)	0.07963 (8)	0.28150 (16)	0.0457 (5)	
H9	−0.0566	0.1152	0.2548	0.055*	
C10	−0.1454 (4)	0.03966 (8)	0.31629 (19)	0.0565 (6)	
H10A	−0.0694	0.0026	0.3349	0.085*	
H10B	−0.2851	0.0349	0.2577	0.085*	
H10C	−0.1964	0.0556	0.3787	0.085*	
C11	0.1175 (4)	0.05314 (12)	0.18821 (17)	0.0678 (7)	
H11A	0.2218	0.0800	0.1612	0.102*	
H11B	−0.0208	0.0440	0.1312	0.102*	
H11C	0.2068	0.0182	0.2119	0.102*	
C12	1.1480 (3)	0.21675 (9)	0.59454 (15)	0.0477 (5)	
H12A	1.2862	0.1926	0.5913	0.072*	
H12B	1.1371	0.2222	0.6693	0.072*	
H12C	1.1672	0.2538	0.5621	0.072*	
C13	0.6056 (3)	0.30757 (6)	0.38532 (12)	0.0258 (3)	
H13	0.4401	0.2915	0.3746	0.031*	
C14	0.6805 (3)	0.32676 (7)	0.50149 (12)	0.0368 (4)	
H14A	0.6821	0.2937	0.5496	0.044*	
H14B	0.8441	0.3430	0.5144	0.044*	
C15	0.5051 (4)	0.37148 (8)	0.52512 (15)	0.0466 (5)	
H15A	0.3454	0.3539	0.5191	0.056*	
H15B	0.5590	0.3852	0.5994	0.056*	
C16	0.4846 (4)	0.42147 (8)	0.44964 (18)	0.0556 (5)	
H16A	0.6385	0.4423	0.4624	0.067*	
H16B	0.3596	0.4478	0.4637	0.067*	
C17	0.4210 (4)	0.40189 (8)	0.33352 (16)	0.0510 (5)	
H17A	0.2583	0.3852	0.3185	0.061*	
H17B	0.4198	0.4351	0.2859	0.061*	
C18	0.5991 (3)	0.35795 (7)	0.30975 (13)	0.0369 (4)	
H18A	0.7604	0.3752	0.3195	0.044*	
H18B	0.5505	0.3449	0.2348	0.044*	

### Atomic displacement parameters (Å^2^)

**Table:**

	*U*^11^	*U*^22^	*U*^33^	*U*^12^	*U*^13^	*U*^23^
S1	0.0263 (2)	0.0315 (2)	0.0264 (2)	−0.00097 (14)	0.00923 (16)	−0.00148 (14)
O1	0.0547 (8)	0.0371 (7)	0.0309 (6)	0.0125 (6)	0.0067 (6)	0.0045 (5)
O2	0.0258 (6)	0.0519 (7)	0.0481 (7)	−0.0035 (5)	0.0136 (5)	−0.0003 (6)
O3	0.0509 (8)	0.0380 (6)	0.0257 (6)	−0.0039 (5)	0.0114 (5)	−0.0038 (5)
C1	0.0311 (8)	0.0296 (8)	0.0290 (8)	0.0061 (6)	0.0079 (7)	−0.0002 (6)
C2	0.0386 (9)	0.0239 (8)	0.0316 (8)	0.0059 (6)	0.0137 (7)	0.0014 (6)
C3	0.0381 (9)	0.0259 (8)	0.0353 (8)	0.0028 (6)	0.0106 (7)	0.0048 (6)
C4	0.0419 (10)	0.0261 (8)	0.0460 (10)	0.0020 (7)	0.0177 (8)	0.0024 (7)
C5	0.0652 (13)	0.0259 (9)	0.0494 (11)	−0.0007 (8)	0.0286 (10)	0.0042 (7)
C6	0.0719 (14)	0.0298 (9)	0.0365 (9)	0.0080 (9)	0.0203 (9)	0.0076 (7)
C7	0.0531 (11)	0.0282 (8)	0.0302 (8)	0.0114 (7)	0.0129 (8)	0.0014 (7)
C8	0.0383 (9)	0.0369 (9)	0.0321 (8)	0.0119 (7)	0.0104 (7)	−0.0006 (7)
C9	0.0407 (10)	0.0320 (9)	0.0653 (13)	−0.0056 (8)	0.0131 (9)	0.0057 (8)
C10	0.0537 (12)	0.0407 (11)	0.0843 (16)	−0.0120 (9)	0.0353 (11)	−0.0130 (11)
C11	0.0508 (13)	0.1066 (19)	0.0474 (12)	−0.0300 (13)	0.0135 (10)	−0.0090 (12)
C12	0.0380 (10)	0.0636 (13)	0.0375 (10)	0.0114 (9)	−0.0010 (8)	−0.0016 (9)
C13	0.0226 (7)	0.0271 (7)	0.0280 (8)	−0.0010 (6)	0.0058 (6)	0.0000 (6)
C14	0.0450 (10)	0.0387 (9)	0.0271 (8)	0.0071 (7)	0.0084 (7)	−0.0029 (7)
C15	0.0551 (12)	0.0444 (11)	0.0426 (10)	0.0081 (9)	0.0151 (9)	−0.0105 (8)
C16	0.0661 (14)	0.0319 (10)	0.0688 (14)	0.0063 (9)	0.0139 (11)	−0.0103 (9)
C17	0.0616 (13)	0.0346 (10)	0.0542 (12)	0.0126 (9)	0.0067 (10)	0.0081 (8)
C18	0.0459 (10)	0.0318 (9)	0.0316 (8)	−0.0020 (7)	0.0051 (7)	0.0048 (7)

### Geometric parameters (Å, °)

**Table:**

S1—O3	1.4389 (12)	C10—H10C	0.9800
S1—O2	1.4389 (12)	C11—H11A	0.9800
S1—C1	1.7345 (16)	C11—H11B	0.9800
S1—C13	1.7747 (15)	C11—H11C	0.9800
O1—C8	1.370 (2)	C12—H12A	0.9800
O1—C7	1.383 (2)	C12—H12B	0.9800
C1—C8	1.363 (2)	C12—H12C	0.9800
C1—C2	1.450 (2)	C13—C18	1.524 (2)
C2—C3	1.389 (2)	C13—C14	1.527 (2)
C2—C7	1.396 (2)	C13—H13	1.0000
C3—C4	1.395 (2)	C14—C15	1.526 (2)
C3—H3	0.9500	C14—H14A	0.9900
C4—C5	1.403 (2)	C14—H14B	0.9900
C4—C9	1.514 (3)	C15—C16	1.510 (3)
C5—C6	1.370 (3)	C15—H15A	0.9900
C5—H5	0.9500	C15—H15B	0.9900
C6—C7	1.371 (3)	C16—C17	1.525 (3)
C6—H6	0.9500	C16—H16A	0.9900
C8—C12	1.475 (3)	C16—H16B	0.9900
C9—C10	1.515 (2)	C17—C18	1.524 (3)
C9—C11	1.515 (3)	C17—H17A	0.9900
C9—H9	1.0000	C17—H17B	0.9900
C10—H10A	0.9800	C18—H18A	0.9900
C10—H10B	0.9800	C18—H18B	0.9900
			
O3—S1—O2	118.13 (7)	C9—C11—H11C	109.5
O3—S1—C1	107.55 (7)	H11A—C11—H11C	109.5
O2—S1—C1	109.19 (8)	H11B—C11—H11C	109.5
O3—S1—C13	108.30 (7)	C8—C12—H12A	109.5
O2—S1—C13	108.69 (7)	C8—C12—H12B	109.5
C1—S1—C13	104.09 (7)	H12A—C12—H12B	109.5
C8—O1—C7	107.01 (12)	C8—C12—H12C	109.5
C8—C1—C2	107.65 (14)	H12A—C12—H12C	109.5
C8—C1—S1	126.16 (13)	H12B—C12—H12C	109.5
C2—C1—S1	126.13 (12)	C18—C13—C14	110.67 (13)
C3—C2—C7	118.78 (15)	C18—C13—S1	109.58 (11)
C3—C2—C1	136.71 (15)	C14—C13—S1	112.41 (11)
C7—C2—C1	104.49 (15)	C18—C13—H13	108.0
C2—C3—C4	119.51 (15)	C14—C13—H13	108.0
C2—C3—H3	120.2	S1—C13—H13	108.0
C4—C3—H3	120.2	C15—C14—C13	109.55 (14)
C3—C4—C5	118.71 (16)	C15—C14—H14A	109.8
C3—C4—C9	119.30 (15)	C13—C14—H14A	109.8
C5—C4—C9	121.93 (16)	C15—C14—H14B	109.8
C6—C5—C4	123.01 (17)	C13—C14—H14B	109.8
C6—C5—H5	118.5	H14A—C14—H14B	108.2
C4—C5—H5	118.5	C16—C15—C14	112.06 (16)
C5—C6—C7	116.56 (16)	C16—C15—H15A	109.2
C5—C6—H6	121.7	C14—C15—H15A	109.2
C7—C6—H6	121.7	C16—C15—H15B	109.2
C6—C7—O1	126.13 (15)	C14—C15—H15B	109.2
C6—C7—C2	123.41 (17)	H15A—C15—H15B	107.9
O1—C7—C2	110.46 (15)	C15—C16—C17	111.05 (15)
C1—C8—O1	110.38 (15)	C15—C16—H16A	109.4
C1—C8—C12	134.37 (17)	C17—C16—H16A	109.4
O1—C8—C12	115.25 (15)	C15—C16—H16B	109.4
C4—C9—C10	115.43 (17)	C17—C16—H16B	109.4
C4—C9—C11	110.26 (16)	H16A—C16—H16B	108.0
C10—C9—C11	108.82 (16)	C18—C17—C16	111.48 (15)
C4—C9—H9	107.3	C18—C17—H17A	109.3
C10—C9—H9	107.3	C16—C17—H17A	109.3
C11—C9—H9	107.3	C18—C17—H17B	109.3
C9—C10—H10A	109.5	C16—C17—H17B	109.3
C9—C10—H10B	109.5	H17A—C17—H17B	108.0
H10A—C10—H10B	109.5	C17—C18—C13	109.17 (14)
C9—C10—H10C	109.5	C17—C18—H18A	109.8
H10A—C10—H10C	109.5	C13—C18—H18A	109.8
H10B—C10—H10C	109.5	C17—C18—H18B	109.8
C9—C11—H11A	109.5	C13—C18—H18B	109.8
C9—C11—H11B	109.5	H18A—C18—H18B	108.3
H11A—C11—H11B	109.5		
			
O3—S1—C1—C8	149.94 (14)	C2—C1—C8—O1	0.67 (18)
O2—S1—C1—C8	20.63 (17)	S1—C1—C8—O1	177.99 (11)
C13—S1—C1—C8	−95.29 (15)	C2—C1—C8—C12	−178.92 (18)
O3—S1—C1—C2	−33.21 (15)	S1—C1—C8—C12	−1.6 (3)
O2—S1—C1—C2	−162.53 (13)	C7—O1—C8—C1	−0.73 (17)
C13—S1—C1—C2	81.56 (14)	C7—O1—C8—C12	178.94 (14)
C8—C1—C2—C3	−179.01 (18)	C3—C4—C9—C10	−164.47 (16)
S1—C1—C2—C3	3.7 (3)	C5—C4—C9—C10	18.3 (2)
C8—C1—C2—C7	−0.33 (17)	C3—C4—C9—C11	71.7 (2)
S1—C1—C2—C7	−177.66 (12)	C5—C4—C9—C11	−105.5 (2)
C7—C2—C3—C4	−0.6 (2)	O3—S1—C13—C18	−63.30 (12)
C1—C2—C3—C4	177.97 (17)	O2—S1—C13—C18	66.20 (12)
C2—C3—C4—C5	1.2 (2)	C1—S1—C13—C18	−177.53 (11)
C2—C3—C4—C9	−176.17 (15)	O3—S1—C13—C14	173.20 (11)
C3—C4—C5—C6	−1.0 (3)	O2—S1—C13—C14	−57.30 (13)
C9—C4—C5—C6	176.28 (18)	C1—S1—C13—C14	58.97 (13)
C4—C5—C6—C7	0.1 (3)	C18—C13—C14—C15	58.75 (18)
C5—C6—C7—O1	−178.51 (16)	S1—C13—C14—C15	−178.37 (12)
C5—C6—C7—C2	0.5 (3)	C13—C14—C15—C16	−56.1 (2)
C8—O1—C7—C6	179.64 (16)	C14—C15—C16—C17	54.4 (2)
C8—O1—C7—C2	0.51 (17)	C15—C16—C17—C18	−55.1 (2)
C3—C2—C7—C6	−0.3 (2)	C16—C17—C18—C13	57.4 (2)
C1—C2—C7—C6	−179.27 (16)	C14—C13—C18—C17	−59.57 (18)
C3—C2—C7—O1	178.86 (14)	S1—C13—C18—C17	175.92 (12)
C1—C2—C7—O1	−0.11 (17)		

### Hydrogen-bond geometry (Å, °)

**Table:**

Cg is the centroid of the C2–C7 benzene ring.

**Table:**

*D*—H···*A*	*D*—H	H···*A*	*D*···*A*	*D*—H···*A*
C13—H13···O2^i^	1.00	2.33	3.3030 (19)	165
C14—H14A···O3^ii^	0.99	2.58	3.424 (2)	143
C10—H10C···Cg^i^	0.98	2.71	3.548 (2)	144

Symmetry codes: (i) *x*−1, *y*, *z*; (ii) *x*, −*y*+1/2, *z*+1/2.
